# The Hidden Burden of Food Hypersensitivity: Exploring Perceived Stress and Fatigue Symptoms in Children with Food Allergies and Intolerances

**DOI:** 10.3390/nu18091371

**Published:** 2026-04-27

**Authors:** Roxana Maria Martin-Hadmaș, Teodora Muscalu, George Mihăiță Gavra, Ștefan Adrian Martin

**Affiliations:** 1 Department of Community Nutrition and Food Safety, “George Emil Palade” University of Medicine, Pharmacy, Science and Technology of Târgu Mures, Gheorghe Marinescu 38, 540139 Târgu Mures, Romania; roxana.hadmas@umfst.ro (R.M.M.-H.);; 2 Center for Advanced Medical and Pharmaceutical Research, “George Emil Palade” University of Medicine, Pharmacy, Science and Technology of Târgu Mures, Gheorghe Marinescu 38, 540139 Târgu Mures, Romania; george.gavra@umfst.ro

**Keywords:** food allergy, food intolerance, perceived stress, quality of life

## Abstract

*Introduction*: Food allergies and intolerances represent a growing pediatric health concern. While stress-related outcomes have received increasing attention, fatigue symptoms in children with food hypersensitivity remain insufficiently characterized. The aim of the study was to evaluate perceived stress and fatigue in children with food hypersensitivity. *Material and Methods:* We conducted an observational cross-sectional study including 339 children aged 1–18 years with specialist-confirmed food allergy, food intolerance, or mixed pathology. Data were collected through structured parent-reported interviews incorporating the Perceived Stress Questionnaire (PSQ) and the Fatigue Assessment Scale (FAS). *Results:* Children with food allergy had significantly higher perceived stress and fatigue scores compared to those with food intolerance (*p* < 0.05), with the highest levels observed in the mixed pathology group. Both perceived stress and fatigue scores increased with age, with adolescents showing the highest values. A moderate positive correlation was identified between stress and fatigue (r = 0.49, *p* < 0.0001). In multivariable analyses, higher stress and fatigue scores were significantly associated with age and diagnostic category, including mixed pathology, after adjustment for sex and anthropometric indicators. Higher scores were also associated with the presence of multiple clinical symptoms, such as sleep disturbances and concentration difficulties. *Conclusions:* Food hypersensitivity in children is associated with a significant psychological burden characterized by elevated perceived stress and fatigue, particularly in adolescents and in those with more complex diagnostic profiles. These findings highlight the importance of multidisciplinary management strategies integrating accurate diagnosis, nutritional counseling, and psychosocial support in order to address the broader impact of pediatric food hypersensitivity.

## 1. Introduction

Food allergies and intolerances represent an increasing burden in pediatric practice, with significant medical, nutritional, and psychosocial implications. Prevalence has risen steadily over the past decade, currently affecting an estimated 5–8% of children worldwide [1,2]. However, this global estimate masks substantial geographic variability and methodological heterogeneity. Prevalence differs across countries/regions and is strongly influenced by the case definition and assessment method (e.g., self-reported vs. physician-diagnosed vs. oral food challenge-confirmed), whereas robust population-based data remain scarce in many low- and middle-income regions [3].

Food allergy encompasses clinically distinct phenotypes with different mechanisms and diagnostic approaches, and it may also coexist with other atopic conditions, further increasing the complexity of clinical management [4]. Recent burden estimates report that asthma is present in a substantial proportion of individuals with food allergy, while several published papers of children with asthma report food allergy [5,6]. On the other hand, food intolerances do not involve the immune system [7]. They are typically characterized by dose-dependent digestive discomfort or other non-specific symptoms following ingestion of certain foods [8]. Understanding these distinctions is important, particularly in childhood and adolescence, where diagnostic challenges and misclassification can significantly impact health and quality of life. As a consequence, many children undergo unnecessary or prolonged elimination of staple foods, placing them at considerable risk for impaired growth and weight development, nutritional deficiencies, disturbances in gut microbiota, and immune system alterations [9,10].

Beyond their physiological consequences, dietary restrictions, coupled with the unpredictability of allergic reactions, may contribute to chronic stress in both children and their parents [11]. Recent evidence suggests that pediatric food allergy is associated with a substantial psychological burden, including anticipatory anxiety, hypervigilance, and social or educational exclusion [12]. These difficulties are frequently accompanied by altered sleep patterns, impaired concentration, and somatic symptoms, which may further exacerbate stress and fatigue [13,14]. This cumulative psychophysiological burden, shaped by dietary restrictions, unpredictability of reactions, and atopic comorbidity, may activate the hypothalamic–pituitary–adrenal (HPA) axis, which modulates both immune reactivity and perceived fatigue. Yet, fatigue symptoms in food-sensitive children remain insufficiently studied and are rarely examined alongside perceived stress or compared across clinically relevant subgroups (food allergy vs. intolerance; IgE-mediated vs. non-IgE/mixed phenotypes). The limited available studies on chronic fatigue in pediatric allergy are heterogeneous and frequently lack rigorous control of diagnostic accuracy [14,15].

The present study aimed to evaluate perceived stress and fatigue symptoms in a cohort of children diagnosed with food allergies and/or food intolerances and to explore whether these outcomes differ according to the type and complexity of food hypersensitivity. We hypothesized that children presenting mixed pathology (coexisting food allergy and intolerance) would report higher levels of perceived stress and fatigue compared with those presenting a single diagnostic category. In addition, we explored the associations between stress, fatigue, anthropometric parameters, and selected clinical symptoms.

## 2. Materials and Methods

We conducted an observational, cross-sectional analytical study over 13 months (September 2024–October 2025) on a sample of 339 participants from Romania. The study was conducted in accordance with the Declaration of Helsinki and approved by the “George Emil Palade” University of Medicine, Pharmacy, Science and Technology of Târgu Mureș Ethics Committee, no. 3332 on 19 August 2024.

### 2.1. Recruitment and Sampling

A total of 450 eligible families were approached during routine follow-up visits in the allergology/clinical immunology outpatient setting. Of these, 339 accepted to participate, resulting in a participation rate of 75.3% (339/450). Potential participants were identified by the treating specialist according to the predefined eligibility criteria. Parents/legal guardians were invited to participate after receiving standardized study information. A total of 339 families provided consent and completed the structured interview. All data were obtained directly from parents via a structured interview.

Eligibility was determined before inclusion (screening at the time of recruitment), and only children meeting the criteria were invited to participate. Accordingly, the analytical study sample consisted of 339 children aged 1–18 years who met the predefined eligibility criteria. Inclusion criteria were: age between 1 and 18 years; permanent residence in Romania; regular follow-up by a specialist in allergology and clinical immunology; and a documented diagnosis of at least one food allergy and/or food intolerance. Exclusion criteria were: lack of at least one specialist consultation for diagnosis or confirmation of the condition; food intolerance diagnosed solely based on IgG antibody testing; diagnosis established in Romania but current residence in another country; and the presence of other chronic medical, neurological, or psychiatric conditions unrelated to food allergy or intolerance that could confound the assessment of stress or fatigue. Conditions commonly associated with food allergy, such as asthma, atopic dermatitis, and allergic rhinitis, were not systematically excluded unless they were deemed by the attending specialist to significantly impact sleep, fatigue, or stress independently of the food hypersensitivity.

### 2.2. Diagnostic Definitions and Clinical Verification (Food Allergy vs. Food Intolerance)

The comprehensive diagnostic process for all study participants was rigorously conducted by specialist physicians in allergology–immunology or pediatric gastroenterology, strictly adhering to national and international protocols. This stage was completed before their integration into the study, ensuring that all subjects presented with clinically verified and certain diagnoses of adverse food reactions based on established medical expertise. Diagnoses of food allergies were precisely differentiated between IgE-mediated and non-IgE-mediated reactions. For IgE-mediated food allergies (e.g., cow’s milk protein allergy), diagnoses relied on detailed medical history, skin prick tests, and/or serum-specific IgE levels, often confirmed by controlled food challenges or elimination/reintroduction diets under medical supervision. For non-IgE-mediated food allergies, diagnosis was primarily clinical, based on symptom presentation (e.g., gastrointestinal symptoms occurring hours to days after exposure) and confirmed through elimination and reintroduction diets.

For non-celiac gluten/wheat sensitivity, the diagnostic protocol involved an initial gluten/wheat elimination diet followed by an oral challenge to confirm symptom recurrence upon reintroduction. Crucially, this process included the rigorous exclusion of celiac disease (via serological tests like IgA, tTG-IgG, and deamidated gliadin peptide-IgG) and wheat allergy (via skin prick tests and specific IgE to wheat). Lactose intolerance diagnosis was confirmed using the hydrogen breath test, established on a positive result and symptomatic correlation with lactose ingestion, following a prior elimination diet. Furthermore, the initial diagnostic process for all conditions consistently included the rigorous exclusion of other gastrointestinal pathologies, such as Helicobacter pylori infection (using C-urea breath test or fecal antigen test) and inflammatory bowel diseases (via medical history, clinical examination, and inflammatory markers). This meticulous approach ensured that all diagnoses of food allergy or intolerance were firmly and accurately established by the treating physicians, ruling out other potential causes for symptoms.

In this study, the term food hypersensitivity is used as a general descriptor referring to any of the following: food allergy, food intolerance, or mixed pathology. This term does not replace the individual diagnostic categories, which are analyzed separately.

### 2.3. Data Collection

The data were collected by a member of the research team, assigned on a rotating basis, using a structured interview and a standardized guide developed by the research team to ensure consistent wording and ordering of questions across participants. Because the study included a wide pediatric age range (1–18 years), questionnaire responses were primarily provided by parents or legal guardians acting as proxy respondents. This approach ensured feasibility and consistency across age groups, particularly for younger children who are unable to reliably self-report psychological symptoms. The child was not required to complete the questionnaire independently; when available, the child could be present and provide input on subjective experiences (e.g., stress- and fatigue-related symptoms), while the parent/legal guardian provided the final recorded response within the same interview framework.

### 2.4. Questionnaire Administration

The interview was based on a 67-item questionnaire specifically developed for this project and conceived as an integrated assessment tool combining (i) project-specific items addressing food allergies and/or intolerances and related dietary management and (ii) the complete, validated 30-item Perceived Stress Questionnaire (PSQ) and the complete, validated 10-item Fatigue Assessment Scale (FAS) to asses stress- and fatigue-related symptoms [16,17]. The interview guide covered three main domains: (1) demographic and anthropometric characteristics, (2) clinical and dietary features related to documented food allergies and/or intolerances (foods involved, typical reaction severity, and dietary modifications implemented by the family), and (3) stress- and fatigue-related symptoms. During the interviews, parents reported demographic and anthropometric information, including each child’s date of birth, current weight and height, and weight-for-height progression over the previous 6–12 months. Anthropometric data were parent-reported, based on recent clinical measurements, and were not directly obtained. The reported values were subsequently processed using the Peditools platform in order to derive weight-for-age, length-for-age, and weight-for-length/BMI-for-age percentiles according to WHO/CDC growth charts. Percentile values of 0 or 100 represent valid outputs generated by the Peditools calculator [18] and do not indicate missing data.

School enrollment status and educational stage were parent-reported and recorded as (i) not yet integrated in the education system or (ii) currently integrated, along with the current education level. For interpretability, education levels were categorized according to the Romanian education structure, using typical age ranges: early childhood education (1–3 years), preschool education (3–6 years), primary education (6–10/11 years), middle school education (10/11–14/15 years), and high school education (14/15–18/19 years).

### 2.5. Statistical Analysis

Data were statistically analyzed using GraphPad Prism version 9.3.0. The significance level was set at a 95% confidence interval (95% CI). Descriptive statistics were presented as mean ± standard deviation, median and coefficient of variation (CV%). Bivariate associations were assessed using the Spearman test, while comparisons among two or more variables were evaluated using Mann–Whitney or Kruskal–Wallis tests.

To identify the independent predictors of perceived stress and fatigue, multivariable linear regression models were constructed, adjusting for age, sex, and weight for length/BMI for age percentiles as covariates. The diagnostic category was treated as a multicategorical predictor using dummy coding, with the “food intolerance” group serving as the reference category. Although the residuals of the models exhibited a non-normal distribution (D’Agostino–Pearson test), the large sample size (n = 339) provided sufficient robustness for the linear estimates. Model stability and the absence of multicollinearity were confirmed using the Variance Inflation Factor (VIF), with all values ranging between 1.034 and 1.471, indicating stable coefficient estimates. The explanatory power of the models was evaluated using the coefficient of determination (R^2^).

To account for multiple comparisons and control the False Discovery Rate, *p*-values from all applied tests and multivariate linear regression models were adjusted using the Benjamini–Hochberg procedure.

## 3. Results

### 3.1. Sociodemographic and Clinical Characteristics of the Sample

The sample included 58.41% male subjects (n = 198) and 41.59% female subjects (n = 141). Urban representation was higher (77.29%, n = 262) compared to rural (22.71%, n = 77). Anthropometric measures showed substantial variability, reflecting the heterogeneity of the sample, as further detailed in Table 1.

Of the entire studied sample, 89.97% (n = 305) were integrated into the education system. Their distribution by education level was as follows: 33.63% (n = 114) in preschool and early childhood education, 32.45% (n = 110) in the primary system, 11.5% (n = 39) in middle school, and 11.21% (n = 38) in high school. Sports activity was reported by 58.5% of participants as follows: 23.3% (n = 79) in endurance sports, 8.84% (n = 30) in team sports, and 26.25% (n = 89) in other individual sports.

A total of 43.36% (n = 147) had at least one confirmed food allergy, and 28.61% (n = 97) had food intolerance, while mixed pathology (both allergy and intolerance) was present in 28.02% (n = 95), as further detailed in Table 2.

### 3.2. Clinical Symptom Profile, Perceived Stress, and Fatigue

Symptoms differed between allergies and intolerances. Children with food allergy more frequently reported rash (28.91%), overall cutaneous manifestations (49.26%), and respiratory symptoms (20.35%), whereas intolerances were dominated by gastrointestinal symptoms, including nausea, vomiting, and gastroesophageal reflux (23.59%), along with changes in bowel habits (28.02%). Other symptoms, such as headache or irritability, were reported in less than 10% of participants.

Of the study sample, 4.42% of subjects reported no signs of perceived stress, whereas 27.73% reported changes in eating behavior, 29.2% impulsivity, 13.27% aggressive behavior, and 25.37% sleep disorders. Regarding symptoms associated with fatigue, 23.01% reported concentration difficulties, 10.62% headache, migraine, or muscle pain, 23.89% shallow sleep, and 12.86% exhaustion after physical, mental, or emotional exertion.

**Figure 1:**
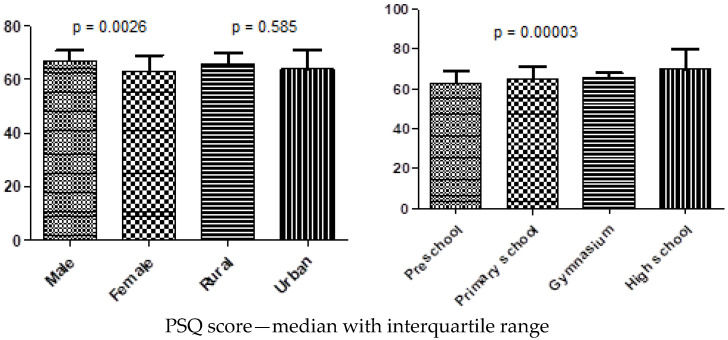

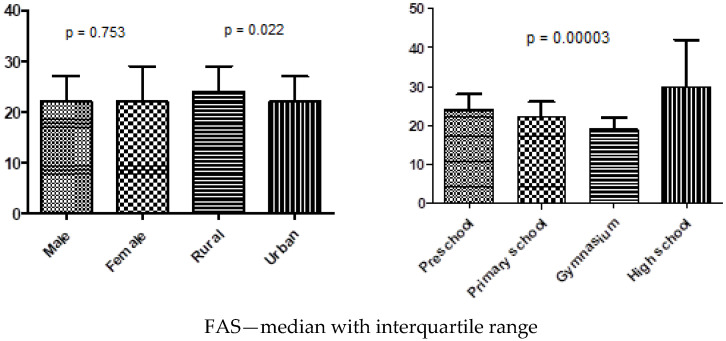
PSQ/FAS score in different groups.

Physical activity failed to correlate with stress (initial *p* = 0.5005; adjusted *p* = 0.5005) or fatigue (initial *p* = 0.0641; adjusted *p* = 0.08546). However, as shown in Figure 1, both perceived stress (PSQ) and fatigue (FAS) scores differed across the analyzed diagnostic categories, with the highest median values observed in children with mixed pathology and lower values in those with isolated food intolerance, indicating a greater psychological burden in children with more complex food hypersensitivity profiles.

FAS scores were statistically different (*p* < 0.001), the post hoc analyses showing an adjusted *p* value of 0.002 to the difference between mixed pathology–intolerance groups, 0.0216 between intolerance–allergy groups, and <0.0001 between mixed pathology–allergy groups. PSQ scores were statistically different (*p* = 0.0248), the post hoc analyses showing an adjusted *p* value of 0.0089 to the difference between mixed pathology–intolerance groups, 0.0089 between intolerance–allergy groups, and without significant data between mixed pathology–allergy groups (corrected *p* value = 0.2849).

A positive correlation was observed between PSQ and anthropometric percentiles (weight for age and weight for length/BMI for age), suggesting potential interactions between nutritional status, food accessibility, and psychological stress. In addition, higher PSQ scores were associated with higher FAS scores (r = 0.4856, initial *p* < 0.0001; adjusted *p* < 0.0001), as further detailed in Table 3.

Both the PSQ score and the fatigue score were positively and statistically significantly associated with the frequency of sleep problems, concentration, pain, fatigue, and mood swings (Table 4).

The PSQ score showed significant differences according to the type of food allergy (*p* < 0.0001), with the highest mean value observed in children with allergy to soy and other beans (PSQ = 77), and the lowest mean value being recorded for milk (PSQ = 65.99). Among children with food intolerances, PSQ scores did not differ significantly either according to the culprit food (*p* = 0.4653) or according to the reported symptom profile (*p* = 0.4621). The PSQ scores differed significantly according to allergy-related symptomatology (*p* < 0.0001), ranging from a mean of 66.88 in the case of transit changes, and up to 72.74 in those with respiratory difficulties.

FAS score also differed significantly according to the type of food allergies (*p* = 0.0354) and food intolerances (*p* = 0.0137), with mean values ranging from 22.44 for allergy to various vegetables/fruits to 31.5 for soy/other legumes, and from 22.5 for egg intolerance to 31.33 for soy/other legumes. Chronic fatigue scores did not differ according to symptomatology reported for food intolerances (*p* = 0.9388), but they differed significantly across allergy-related symptoms (*p* = 0.0355), with mean values ranging from 22.46 for skin rashes to 25.39 for nausea, vomiting, and gastroesophageal reflux.

### 3.3. Independent Predictors of Perceived Stress and Fatigue: Multivariable Analysis

The multivariable model for perceived stress was statistically significant (initial *p* = 0.0002, adjusted *p* = 0.001). Age emerged as a dominant independent predictor of stress levels (β = 0.406, initial *p* = 0.0002, adjusted *p* = 0.001). While a single food allergy did not significantly differ from the reference group (initial *p* = 0.1183, adjusted *p* = 0.155), the mixed pathology group maintained a significantly higher stress burden (β = 3.318, initial *p* = 0.0144, adjusted *p* = 0.036) even after adjusting for age and sex.

In the case of FAS, both age (β = 0.404, initial *p* < 0.0001, adjusted *p* < 0.0001) and weight for length/BMI for age percentiles (β = 0.0248, initial *p* = 0.0170, adjusted *p* = 0.036) were identified as significant positive predictors, indicating that fatigue levels increase with advancing biological maturity and higher nutritional status indicators. In contrast, participants from the food allergy group presented significantly lower fatigue scores compared with the reference group (β = −2.830, initial *p* = 0.0010, adjusted *p* = 0.003). Conversely, the mixed pathology group remained the most affected phenotype, exhibiting significantly higher fatigue levels (β = 2.202, initial *p* = 0.0195, adjusted *p* = 0.036), as detailed in Table 5.

## 4. Discussion

The present study confirms and extends existing evidence that food allergies and intolerances in children are associated with increased perceived stress and fatigue symptoms. Allergies to common triggers such as milk, egg, peanut, soy, and tree nuts have repeatedly been associated with heightened vigilance and fear of accidental exposure, increased parental burden, and diminished quality of life [19,20,21]. Several studies also indicate that the anticipatory anxiety surrounding severe reactions contributes more to the psychological burden than the reactions themselves [22,23,24,25]. However, because of the cross-sectional design of this study, the observed relationships should be interpreted as associations rather than causal effects. It is also important to acknowledge that perceived stress in children and adolescents is multifactorial and may be influenced by developmental stage, school-related demands, family context, and other psychosocial factors not captured in the present dataset. While our findings suggest links between food hypersensitivity, perceived stress, and fatigue-related symptoms, the temporal direction of these relationships cannot be determined. Therefore, the interpretations presented below are intended to generate hypotheses for future longitudinal research rather than establish causal mechanisms.

### 4.1. Psychological and Clinical Correlates of Stress and Fatigue

Our observation that allergies to soy and beans are associated with disproportionate psychological strain is consistent with recent data on the challenges associated with widespread allergens. The ubiquity of soy derivatives in processed foods greatly increases parental vigilance and feelings of loss of control, as reliable identification of the allergen is often difficult due to labeling gaps and cross-contamination risks [26]. In contrast, allergens for which suitable substitutes are readily available tend to be associated with lower stress levels. Taken together, these findings support the notion that allergen prevalence and its integration into the general food environment are key determinants of perceived stress in families managing pediatric food allergies [27,28].

A pivotal finding that emerged from our adjusted analysis is the predominant role of age as an independent predictor of perceived stress (β = 0.406, initial *p* = 0.0002, adjusted *p* = 0.001). Therefore, although food hypersensitivity may contribute to psychological burden, the observed stress levels cannot be attributed exclusively to the allergic or intolerance profile. However, multiple studies demonstrate that the adolescent brain’s increased sensitivity to stress, combined with growing autonomy in food-related decision-making, creates a period of heightened vulnerability [26]. Our results mirror other findings showing that adolescents with chronic conditions may have elevated stress reactivity and somatic fatigue, particularly when symptom unpredictability is high [29,30,31,32]. While our univariate analysis initially suggested broad differences between groups, the multivariable model indicates that much of the observed stress in our cohort is a function of developmental maturity and the increasing social complexity associated with managing food restrictions. However, the mixed pathology group maintained a significantly higher stress burden compared to the intolerance-only reference group (β = 3.318, initial *p* = 0.0144, adjusted *p* = 0.036), even after adjusting for age. This finding supports a “cumulative burden” hypothesis: children managing overlapping sensitivities face the acute, life-threatening vigilance required for IgE-mediated allergies alongside the chronic gastrointestinal strain of intolerances.

The strong associations observed between sleep disturbances, concentration problems, dizziness, and musculoskeletal pain with both PSQ and FAS scores are consistent with neuroimmune fatigue models [26]. In these models, chronic stress induces hypothalamic–pituitary–adrenal axis dysregulation, alters sleep architecture, and promotes cytokine-mediated fatigue. Similarly, the existing literature identifies sleep disruption as a mediator between allergic disease severity and cognitive fatigue in children. Our findings expand this data by showing that even mild-to-moderate food sensitivities can produce comparable symptom clusters, emphasizing the broad impact of chronic vigilance and physiological dysregulation [33,34]. This pattern suggests that psychological and physiological factors are intricately linked, with chronic stress impacting sleep quality, cognitive function, and somatic symptoms, which in turn can exacerbate fatigue. The exact mechanisms involve complex neuro-immune interactions that modulate subjective experiences of well-being.

Compared to previous studies focusing primarily on generic quality of life measures, our data provide a more detailed differentiation between the psychological impacts of allergies and intolerances. This distinction is rarely explored, as most international reports examine food sensitivities collectively, potentially obscuring clinically meaningful differences. In this regard, we separately quantified perceived stress and chronic fatigue using PSQ and FAS and directly compared scores across children with IgE-mediated allergy, food intolerance, and mixed pathology, allowing diagnosis- and allergen-specific patterns of psychological burden to emerge. This underscores the importance of precise diagnosis and patient education regarding the nature of their food reactions [20,21].

The lack of a protective effect of physical activity on stress or fatigue scores in our cohort contrasts with the general literature, in which exercise typically reduces stress and improves emotional resilience. However, our findings are not isolated. Recent work suggests that exercise-induced mast cell activation, combined with dietary inadequacies and nutritional risks in children following restrictive diets, may contribute to increased post-exertional symptoms and fatigue. Furthermore, chronic illnesses can lead to fatigue that is not alleviated by physical activity, or even exacerbated by it, particularly in conditions related to immune dysfunction. Although we did not directly assess immunological markers in our cohort, this study adds nuance by demonstrating that these patterns can persist even in non-athletic children with food sensitivities, suggesting that the underlying physiological and psychological burden may override the typical benefits of physical activity [35,36,37].

The positive relationship observed between anthropometric percentiles and stress expands an underexplored dimension in pediatric allergy research. While previous studies have highlighted that food restrictions may impair growth, the opposite association, where a higher anthropometric percentile is linked to higher stress, has received little attention. Regarding fatigue, our study identified age and weight for length/BMI for age as significant independent predictors (β = 0.404, initial *p* < 0.0001, adjusted *p* < 0.0001 and β = 0.0248, initial *p* = 0.0170, adjusted *p* = 0.036, respectively). Possible explanations include increased caloric needs in growing children with allergies, heightened parental and child awareness of dietary limitations and the vigilance required to ensure adequate nutrition, the economic burden associated with ensuring allergen-free foods, and stress-related eating behaviors mediated by cortisol. However, it is important to note that our dataset does not contain specific variables to directly test these hypotheses (e.g., family income, precise dietary intake, observed eating behaviors). Therefore, these explanations remain theoretical and warrant further investigation with specific data points. Our findings encourage further investigation of how nutritional status interacts with psychological responses in food-sensitive children, moving beyond a sole focus on growth restriction [38,39].

The relatively large variability observed in anthropometric percentiles likely reflects the wide developmental range of the cohort (1–18 years). Although age-adjusted percentiles were used to standardize anthropometric indicators, the inclusion of children at very different developmental stages may still introduce substantial biological heterogeneity. This variability should therefore be considered when interpreting the observed associations between anthropometric parameters and perceived stress.

Notably, we observed a “fatigue paradox”: single food allergy was associated with significantly lower fatigue levels compared to the intolerance group (β = −2.830, initial *p* = 0.0010, adjusted *p* = 0.003). This may be attributed to the persistent, daily nature of symptoms in children with food intolerances, which can lead to chronic somatic exhaustion, unlike the more episodic nature of simple food allergies. However, for those suffering from both conditions, fatigue levels remain significantly elevated (β = 2.202, initial *p* = 0.0195, adjusted *p* = 0.036), reinforcing the need for comprehensive nutritional and psychological monitoring in this subgroup.

Overall, our results support and extend the existing literature by providing a more detailed clinical profile of stress and fatigue symptoms in children with food hypersensitivity. The strong correlation between PSQ and FAS observed in our cohort reinforces the interconnected nature of psychological and physiological stress responses. Prior studies have described similar interactions, suggesting that chronic stress promotes immune activation, autonomic imbalance, and cognitive strain, ultimately amplifying subjective fatigue. Our findings confirm these mechanisms in a pediatric allergy population and highlight the importance of early recognition and intervention [40,41].

### 4.2. Limitations and Future Directions

This study identifies several areas where current evidence remains limited. International cohorts rarely differentiate between specific allergens in terms of psychological impact, and few integrate robust fatigue measures. Our results provide novel insights into food sensitivities, specific stress patterns, and emphasize the need for targeted, multidisciplinary approaches involving allergology, nutrition, psychology, and sleep medicine. While our study utilized a symptom scale to assess fatigue-related manifestations, it is important to note that formal diagnoses of fatigue syndrome typically require specific diagnostic criteria. In addition, the Perceived Stress Questionnaire (PSQ) and Fatigue Assessment Scale (FAS) were originally developed as self-report instruments. In the present study, responses were obtained from parents acting as proxy respondents due to the wide age range of participants. While this approach is common in pediatric research, particularly for younger children, it may not fully capture the child’s subjective psychological experience and may reflect parental distress rather than solely the child’s.

Furthermore, we acknowledge that food intolerance represents a heterogeneous group and may be more difficult to confirm with uniform diagnostic criteria; however, we aimed to reduce misclassification by excluding IgG-based diagnoses and relying on specialist follow-up and clinical elimination–reintroduction patterns.

While our multivariable models explained a modest proportion of the variance in stress (R^2^ = 0.0728) and fatigue (R^2^ = 0.1548), these values are consistent with the multifactorial nature of pediatric psychological health. The identification of stable, independent predictors despite these values underscores the complexity of the psychophysiological response to food hypersensitivities. Although the multivariable models included age, sex, and anthropometric indicators as covariates, other potentially important confounders were not available in the dataset. The broad age range of the cohort (1–18 years) represents an additional limitation, as it includes markedly different developmental stages that may influence both perceived stress and fatigue, particularly when responses are obtained through parent proxies rather than direct self-report. Furthermore, although age was included as a continuous covariate in all regression models and educational stage was used as a developmental proxy, formal age-stratified subgroup analyses were not feasible given the sample size of 339 participants distributed across five educational stages and three diagnostic categories simultaneously, which would result in cell sizes too small for reliable statistical inference.

In addition, adolescents may inherently report higher perceived stress due to developmental and psychosocial factors, which makes interpreting age-related differences more difficult in a cross-sectional design. Although age was included in the multivariable models and remained a significant independent predictor of both perceived stress and fatigue, residual confounding related to unmeasured psychosocial stressors cannot be excluded. Variables such as socioeconomic status, parental psychological distress, duration of diagnosis, and the severity of allergic reactions may also influence perceived stress and fatigue in children with food hypersensitivity. Future studies incorporating these variables would provide a more comprehensive understanding of the determinants of psychological burden in this population.

Recruitment from a tertiary center and urban predominance may limit generalizability, and the absence of a healthy control group restricts direct comparisons to the general population. Further, the cross-sectional nature of our study precludes the establishment of causal relationships and limits our ability to assess symptom progression or the long-term impact of food hypersensitivity. While our findings identify significant associations, longitudinal studies are essential to assess causality. Future research should also include longitudinal assessments to monitor the ‘allostatic load’ induced by chronic dietary management and investigate the impact of allergy-related bullying, a major social stressor that can exacerbate isolation and the depletion of the child’s psychological resources.

## 5. Conclusions

This study suggests that perceived stress (PSQ) and fatigue symptoms (FAS) are associated with diagnostic complexity, developmental age, and selected anthropometric indicators in children with food hypersensitivity (food allergy, food intolerance or mixed pathology—coexisting food allergy and intolerance). In particular, children presenting mixed pathology reported higher levels of both perceived stress and fatigue compared with those presenting a single diagnostic category.

The identified vulnerability of the mixed pathology group underscores the need for a multidisciplinary approach in clinical management. Healthcare providers should move beyond a diagnosis-centric focus to prioritize psychosocial screening and age-appropriate support, particularly for older children and those with multiple dietary restrictions.

Although causal relationships cannot be established due to the cross-sectional design, the findings highlight the potential importance of considering psychological well-being when managing pediatric food hypersensitivity. Multidisciplinary approaches combining accurate diagnosis, nutritional counseling, and psychosocial support may help mitigate the broader burden experienced by affected children and their families.

## Figures and Tables

**Table 1:** Distribution of anthropometric indicators (weight-for-age, length-for-age, and weight-for-length percentiles) in the study sample.

	Weight for Age	Length for Age	Weight for Length/ BMI for Age
**Min**	0	0	0
**Median (IQR)**	44 (16–81)	53 (15–86)	35.7 (9.1–72.80)
**Mean ± SD**	46.42 ± 33.64	49.02 ± 35.8	43.68 ± 35.8
**Max**	100	100	100

Values of 0 and 100 represent actual percentile outputs generated by the Peditools calculator based on WHO/CDC growth charts and do not indicate missing data.

**Table 2:** Distribution of food allergy, food intolerance, and mixed pathology in the study sample.

	Food Allergy		Food Intolerance	
	%	No.	%	No.
Total	43.36	147	28.61	97
Milk and milk compounds	43.07	146	28.61	97
Eggs	22.42	76	6.49	22
Soy and other beans	3.83	13	-	-
Nuts and seeds	19.17	65	0.88	3
Wheat/gluten and other cereals	5.31	18	22.96	78
Fish and seafood	7.08	24	2.95	10
Histamine	-	-	15.04	51
Vegetables and fruits	17.69	60	15.33	52

Note: The ‘Total’ row reflects the overall prevalence of allergy, intolerance, and mixed pathology within the cohort. For specific food items, percentages and numbers refer to their prevalence within the respective allergy or intolerance categories. The total number of children with milk and milk compound allergy is a subgroup of the total food allergy population. The term “Histamine” under food intolerance refers to cases where individuals presented with symptoms suggestive of histamine intolerance, diagnosed through clinical criteria and response to low-histamine diets, rather than a specific food allergen. This is a recognized clinical entity, characterized by an imbalance between histamine accumulation and degradation capacity. Regarding vegetables and fruits, specific typologies were not differentiated in this study beyond the broad category due to the diverse and individualized nature of reactions to these food groups.

**Table 3:** Association between PSQ/FAS scores and anthropometric parameters.

Parameter I	Parameter II	r Value	95% CI	Initial *p* Value	Adjusted *p* Value
PSQ index	Weight for age	0.2334	0.1270 to 0.3345	<0.0001	<0.0001
	Height for age	0.01369	−0.09613 to 0.1232	0.8017	0.9353
	Weight for length/BMI for age	0.1817	0.07352 to 0.2857	0.0008	0.00186
FAS	Weight for age	0.06958	−0.04040 to 0.1779	0.2013	0.3522
	Height for age	−0.00026	−0.1099 to 0.1094	0.9962	0.9962
	Weight for length/BMI for age	0.05241	−0.05759 to 0.1612	0.336	0.4704
	PSQ index	0.4856	0.3971 to 0.5652	<0.0001	<0.0001

**Table 4:** Association between PSQ/FAS scores and clinical symptoms.

**PSQ Score**					
		**r Value**	**95% CI**	**Initial *p* Value**	**Adjusted** ***p* Value**
Frequency of	Sleep problems	0.3514	0.2514 to 0.4439	<0.0001	<0.0001
	Concentration problems	0.4527	0.3610 to 0.5358	<0.0001	<0.0001
	Muscular pain	0.3961	0.2994 to 0.4847	<0.0001	<0.0001
	Articular pain	0.2747	0.1701 to 0.3731	<0.0001	<0.0001
	Head pain	0.1101	0.003961 to 0.2171	0.0429	0.0446
	Dizziness	0.2632	0.1581 to 0.3625	<0.0001	<0.0001
	Tiredness	0.3216	0.2197 to 0.4166	<0.0001	<0.0001
	Blurred vision	0.2489	0.1432 to 0.3491	<0.0001	<0.0001
	Weakness	0.1915	0.08358 to 0.2950	0.0004	0.00043
	Mood swings	0.3431	0.2426 to 0.4364	<0.0001	<0.0001
	Night sweats	−0.06018	−0.1687 to 0.04982	0.2692	0.2692
	Low body temperature	0.3061	0.2032 to 0.4022	<0.0001	<0.0001
	Irritable bowel	0.312	0.2095 to 0.4077	<0.0001	<0.0001
**FAS**					
		**r Value**	**95% CI**	**Initial *p* Value**	**Adjusted** ***p* Value**
Frequency of	Sleep problems	0.6764	0.6122 to 0.7318	<0.0001	<0.0001
	Concentration problems	0.5376	0.4547 to 0.6112	<0.0001	<0.0001
	Muscular pain	0.5603	0.4801 to 0.6312	<0.0001	<0.0001
	Articular pain	0.4049	0.3089 to 0.4927	<0.0001	<0.0001
	Head pain	0.6119	0.5384 to 0.6762	<0.0001	<0.0001
	Dizziness	0.6444	0.5754 to 0.7043	<0.0001	<0.0001
	Tiredness	0.5591	0.4788 to 0.6301	<0.0001	<0.0001
	Blurred vision	0.6163	0.5434 to 0.6801	<0.0001	<0.0001
	Weakness	0.5422	0.4599 to 0.6153	<0.0001	<0.0001
	Mood swings	0.6072	0.5330 to 0.6721	<0.0001	<0.0001
	Night sweats	0.3896	0.2924 to 0.4788	<0.0001	<0.0001
	Low body temperature	0.4824	0.3936 to 0.5624	<0.0001	<0.0001
	Irritable bowel	0.5874	0.5106 to 0.6548	<0.0001	<0.0001

**Table 5:** Multivariable linear regression models for PSQ and FAS scores.

	PSQ				FAS				VIF
	β	95% CI	Initial *p* Value	Adjusted *p* Value	β	95% CI	Initial *p* Value	Adjusted *p* Value	
**Age**	0.406	0.190 to 0.621	0.0002	0.001	0.404	0.254 to 0.554	<0.0001	<0.0001	1.034
**Sex**	1.468	−0.603 to 3.539	0.1641	0.164	−1.119	−2.560 to 0.322	0.1274	0.155	1.073
**Weight for length/BMI for age**	0.024	−0.005 to 0.053	0.1086	0.155	0.0248	0.004 to 0.045	0.0170	0.036	1.130
**Food allergy**	1.919	−0.492 to 4.331	0.1183	0.155	−2.830	−4.508 to −1.152	0.0010	0.003	1.471
**Mixed pathology**	3.318	0.665 to 5.971	0.0144	0.036	2.202	0.357 to 4.047	0.0195	0.036	1.462

## Data Availability

The original contributions presented in this study are included in the article. Further inquiries can be directed to the corresponding author.

## Abbreviations

The following abbreviations are used in this manuscript:

PSQ	Perceived Stress Questionnaire
FAS	Fatigue Assessment Scale
HPA	Hypothalamic–pituitary–adrenal axis
